# Retrospective comparative study on clinical remission rate and safety of rituximab combined with glucocorticoids versus cyclophosphamide monotherapy in patients with membranous nephropathy

**DOI:** 10.1097/MD.0000000000047093

**Published:** 2026-01-23

**Authors:** Fan Lou, Ming Yao, Hui Chen, Xueqin He

**Affiliations:** a The Pharmacy Department, West China Hospital of Sichuan University, Chengdu, Sichuan Province, China.

**Keywords:** anti-PLA_2_R antibody, cyclophosphamide, primary membranous nephropathy, remission rate, rituximab, safety

## Abstract

This study aimed to compare the clinical efficacy, safety, and immunologic responses between rituximab (RTX) plus glucocorticoids and cyclophosphamide (CTX) monotherapy in patients with primary membranous nephropathy (PMN). A total of 102 patients with biopsy-proven PMN treated at our center between January 2023 and January 2025 were retrospectively analyzed. Patients were divided into the RTX plus glucocorticoid group (RTX group, n = 52) and the CTX monotherapy group (CTX group, n = 50). Baseline characteristics were comparable between groups. Primary endpoints included clinical remission rates (complete + partial) at 6 and 12 months, time to remission, relapse rate and sustained remission, M-type phospholipase A_2_ receptor (PLA_2_R) antibody clearance, safety events, and changes in renal function. At 6 months, the total remission rate was significantly higher in the RTX group than in the CTX group (73.1% vs 54.0%, *P* = .042); at 12 months, remission rates further increased to 84.6% and 66.0%, respectively (*P* = .028). The median time to remission was shorter in the RTX group (3.1 vs 4.5 months, *P* = .011). Among responders, relapse occurred in 9.1% of RTX-treated patients versus 21.2% in the CTX group, with a significantly higher relapse-free survival in the RTX group (*P* = .045). Among PLA_2_R-positive patients, the 12-month antibody clearance rate was higher with RTX (78.3% vs 56.0%, *P* = .035), and clinical remission was more frequent in antibody clearers than in non-clearers (91.2% vs 60.5%, *P* < .01). The overall incidence of adverse events was lower with RTX (13.5% vs 30.0%, *P* = .041), mainly consisting of mild to moderate, reversible reactions. Estimated glomerular filtration rate remained stable in both groups, and no patient progressed to end-stage renal disease. Rituximab combined with glucocorticoids significantly improved remission rates, shortened the time to response, and reduced relapse risk in patients with PMN. Clinical remission was closely associated with PLA_2_R antibody clearance. Compared with CTX, RTX demonstrated a superior safety profile and better tolerability, making it a safe, effective, and mechanistically precise immunological therapy. These findings provide new clinical evidence supporting RTX as a key component of individualized, precision treatment strategies for membranous nephropathy.

## 1. Introduction

Membranous nephropathy (MN) is one of the most common pathological types of nephrotic syndrome in adults, accounting for approximately 30 to 40% of adult cases, with primary membranous nephropathy (PMN) representing the majority.^[1,2]^ The characteristic pathological hallmark of PMN is the subepithelial deposition of immune complexes along the glomerular basement membrane, accompanied by mesangial matrix expansion and glomerular filtration barrier damage.^[3]^ In recent years, accumulating evidence has identified PMN as an autoimmune-mediated glomerular disease strongly associated with autoantibodies against the M-type phospholipase A_2_ receptor (PLA_2_R).^[4,5]^ Anti-PLA_2_R antibodies are detectable in approximately 70 to 80% of patients with PMN, and their titers correlate closely with disease activity, proteinuria level, and therapeutic response. Therefore, these antibodies have been recognized as crucial biomarkers for disease diagnosis, therapeutic monitoring, and relapse prediction.^[6,7]^ The discovery of PLA_2_R antibodies has fundamentally shifted the treatment paradigm of MN from nonspecific immunosuppression toward targeted immunomodulation.

Traditional therapeutic regimens for PMN have primarily relied on the combination of glucocorticoids and immunosuppressants such as cyclophosphamide (CTX). These regimens can induce remission and delay renal function decline in a subset of patients.^[8,9]^ However, this strategy remains limited by several drawbacks: while alkylating agents like CTX effectively suppress immune activity, their onset of action is relatively slow, relapse rates remain high, and significant toxicities – such as bone marrow suppression, increased infection risk, reproductive toxicity, and potential carcinogenicity – often compromise treatment adherence and long-term safety.^[10,11]^ In patients with prolonged disease course, persistent proteinuria, or high antibody titers, conventional regimens often yield suboptimal outcomes. Calcineurin inhibitors (e.g., tacrolimus and cyclosporine A) can transiently reduce proteinuria but are frequently associated with relapse after discontinuation and nephrotoxicity with prolonged use.^[12,13]^ Consequently, identifying a therapeutic strategy that effectively controls immune activation while maintaining an acceptable safety profile has become a central focus in recent MN research.

Rituximab (RTX), a chimeric monoclonal antibody targeting the cluster of differentiation 20 antigen on B lymphocytes, induces selective B-cell depletion, thereby blocking the production of anti-PLA_2_R and other autoantibodies, inhibiting immune complex formation, and alleviating glomerular inflammation and proteinuria.^[14,15]^ Since its first use in refractory MN in 2002, numerous studies have demonstrated that RTX can achieve significant reductions in proteinuria, lower relapse rates, and stabilize renal function.^[16,17]^ Randomized controlled trials conducted abroad have reported remission rates of 60 to 80% within 6 to 12 months, which are comparable to or better than those achieved with conventional immunosuppressive regimens, while exhibiting markedly fewer adverse effects.^[18]^ Moreover, decreases in anti-PLA_2_R antibody titers often precede clinical remission, suggesting a strong linkage between immunologic and clinical responses. Recent cohort studies have further shown that RTX not only induces remission but also prolongs relapse-free survival and improves long-term renal outcomes.^[19,20]^ Nonetheless, variations in RTX efficacy across different populations remain a matter of debate, with factors such as ethnicity, baseline antibody levels, concomitant treatments, and dosage regimens potentially influencing therapeutic outcomes and safety profiles.

Despite growing evidence supporting RTX in MN, most previous studies have been limited by single-arm or small-sample designs, short follow-up durations, and the absence of direct comparisons with traditional regimens. In China, CTX remains widely used as a first-line immunosuppressant, yet comprehensive comparative studies evaluating RTX plus glucocorticoids versus CTX monotherapy in this population remain scarce.^[21,22]^ Furthermore, the temporal dynamics of PLA_2_R antibody clearance and its correlation with clinical remission under different treatment strategies have not been fully elucidated. The present retrospective study, based on real-world data from our center between 2023 and 2025, systematically compared the clinical efficacy, relapse rates, PLA_2_R antibody clearance, safety, and renal function changes between RTX combined with glucocorticoids and CTX monotherapy in biopsy-proven PMN patients. By integrating both clinical and immunologic parameters, this study aimed to provide new evidence for individualized treatment optimization and outcome prediction in PMN.

The novelty of this study lies in 3 main aspects. First, it utilized real-world clinical data with an adequate sample size, including only treatment-naïve patients, thereby minimizing confounding from prior immunosuppressive exposure and objectively reflecting the true comparative efficacy of the 2 regimens. Second, beyond conventional clinical outcomes, this study incorporated dynamic assessment of anti- PLA_2_R antibody levels to explore the association between immunologic remission and clinical response, offering a new perspective for disease monitoring and therapeutic evaluation. Third, by comprehensively analyzing efficacy, safety, and renal function preservation, the study provides an evidence-based framework for balancing the risks and benefits of different immunosuppressive strategies. Collectively, these findings hold significant implications for optimizing treatment strategies and guiding clinical decision-making in MN, while laying the groundwork for future prospective, multicenter studies.

## 2. Method

### 2.1. Study population

This study was approved by the Ethics Committee of Hai’an Hospital of West China Hospital of Sichuan University. This was a single-center, retrospective, controlled study that included 102 patients diagnosed with PMN at our institution between January 2023 and January 2025. All diagnoses were confirmed by renal biopsy and met the diagnostic criteria for PMN outlined in the *Guidelines for the Diagnosis and Treatment of Glomerular Diseases*.

Inclusion criteria were as follows:

1. biopsy-confirmed PMN;
2. baseline 24-hour urinary protein > 3.5 grams and serum albumin < 30 g/L;
3. complete clinical and follow-up data available.

Exclusion criteria included:

1. secondary MN (e.g., systemic lupus erythematosus, hepatitis B virus infection, or malignancy);
2. concomitant primary glomerular diseases;
3. prior use of RTX, CTX, or other immunosuppressants;
4. severe hepatic dysfunction or active infection.

Patients were divided into 2 groups based on their initial treatment strategy: the RTX plus glucocorticoid group (RTX group, n = 52) and the CTX monotherapy group (CTX group, n = 50). All treatment decisions were made by the attending nephrologists in accordance with current clinical guidelines and patient preference.

This study was approved by the Institutional Ethics Committee and conducted in accordance with the principles of the Declaration of Helsinki.

### 2.2. Treatment regimens

Patients in the RTX group received intravenous RTX at a dose of 1 gram per infusion, administered twice at a two-week interval. An additional single-dose consolidation infusion was permitted within 12 months if relapse or anti-PLA_2_R antibody reappearance occurred. All RTX-treated patients concurrently received oral prednisone at 0.5 mg/kg/day for 3 months, followed by a gradual taper until discontinuation.

Patients in the CTX group were treated with CTX at 1.5 to 2.0 mg/kg/day, administered orally or intravenously for a total duration of 6 months, in combination with oral glucocorticoids (initially 0.5 mg/kg/day) tapered to a maintenance dose.

Both groups received an identical glucocorticoid regimen. Oral prednisone was initiated at 0.5 mg/kg/day and maintained for the first 3 months, followed by a standardized tapering schedule (reducing the dose by 5 mg every 2–4 weeks depending on clinical response) until complete withdrawal. No differences in steroid dose, duration, or tapering protocol existed between the 2 groups.

### 2.3. Outcome measures

The primary and secondary outcomes included clinical efficacy, immunologic response, renal function, and safety.

#### 2.3.1. Clinical remission criteria

Complete remission (CR) was defined as 24-hour urinary protein < 0.3 grams, serum albumin ≥ 35 g/L, and normal renal function.

Partial remission (PR) was defined as a ≥50% reduction in proteinuria from baseline with a final value <3.5 g/24 hours, and serum albumin increased by ≥30%.

Patients not meeting the above criteria were classified as having no remission.

#### 2.3.2. Relapse

Defined as recurrence of proteinuria > 3.5 g/24 hours with a concomitant decline in serum albumin ≥ 5 g/L in previously remitted patients.

#### 2.3.3. Anti-PLA_2_R antibody clearance

Anti-PLA_2_R antibody titers were measured using an enzyme-linked immunosorbent assay. Clearance was defined as a titer below the lower detection limit (2 RU/mL).

#### 2.3.4. Renal function evaluation

Estimated glomerular filtration rate (eGFR) was calculated using the CKD-EPI equation. A ≥30% decline from baseline or persistent eGFR < 15 mL/min/1.73 m^2^ was defined as significant renal deterioration, while persistent eGFR < 15 mL/min/1.73 m^2^ for ≥3 months or dialysis dependence was considered end-stage renal disease (ESRD).

#### 2.3.5. Safety assessment

All treatment-related adverse events (AEs) were recorded, including infection, leukopenia, gastrointestinal discomfort, allergic or infusion reactions, and hepatic dysfunction.

### 2.4. Follow-up and evaluation

All patients were followed for 12 months from treatment initiation. The same research team conducted all follow-up evaluations to ensure consistency and reproducibility. Follow-up assessments were performed at baseline and at 1, 3, 6, 9, and 12 months post-treatment. Each visit included evaluation of clinical symptoms, physical examination, laboratory parameters, and adverse drug reactions.

Baseline assessments included medical history, physical examination, laboratory tests, and biopsy review. Follow-up was conducted through a combination of outpatient visits and telephone interviews. Additional assessments were performed earlier if patients experienced increased proteinuria, infection, or other adverse reactions.

At each follow-up, the following parameters were recorded: blood pressure, heart rate, body weight, 24-hour urinary protein (measured by ammonium sulfate turbidimetric method), serum albumin, lipids, creatinine, and eGFR, as well as repeat measurement of anti-PLA_2_R antibody titers.

Therapeutic response was independently evaluated by 2 blinded investigators; any discrepancies were resolved by a third reviewer. Data were double-entered and cross-verified by 2 research assistants, and any outliers were rechecked by the principal investigator. Patients lost to follow-up were contacted via electronic medical records and telephone until study completion. Cases with <80% follow-up adherence were excluded from final analysis.

### 2.5. Statistical analysis

All statistical analyses were performed using SPSS version 26.0 (IBM Corp., Armonk). Continuous variables were expressed as mean ± standard deviation (x̄ ± s). Between-group comparisons were conducted using the independent-samples *t*-test or paired *t*-test, while non-normally distributed data were analyzed with the Mann–Whitney *U* test. Categorical variables were presented as counts and percentages and compared using the χ^2^ test or Fisher exact test as appropriate.

Survival analysis, including relapse-free survival, was performed using the Kaplan–Meier method, and group differences were evaluated with the Log-rank test. A two-tailed *P* value < .05 was considered statistically significant.

## 3. Result

### 3.1. Baseline characteristics of the patients

This study was approved by the Ethics Committee of Hai’an Hospital of West China Hospital of Sichuan University.A total of 102 patients diagnosed with PMN at our center between January 2023 and January 2025 were included in this study. Based on the initial treatment regimen, patients were divided into 2 groups: those receiving RTX combined with glucocorticoids were assigned to the RTX group (n = 52), and those receiving CTX monotherapy were assigned to the CTX group (n = 50). None of the patients had received prior immunosuppressive therapy before enrollment. All treatment strategies were determined by the attending physicians in accordance with current clinical guidelines and patient preferences.

At baseline, there were no statistically significant differences between the 2 groups regarding key demographic parameters, vital signs, baseline renal function, immunologic profiles, lifestyle factors, or comorbidities (*P* > .05), indicating good comparability between the groups. The detailed baseline characteristics are presented in Table 1.

**Table 1:** Baseline characteristics of patients in RTX and CTX groups.

Variable	RTX group (n = 52)	CTX group (n = 50)	Test statistic (*t*/χ^2^)	*P*-value
Age (yr)	45.8 ± 12.3	47.1 ± 11.6	*t* = 0.705	.482
Male (%)	65.4% (34/52)	62.0% (31/50)	χ^2^ = 0.143	.705
Height (cm)	167.9 ± 8.5	168.2 ± 7.9	*t* = 0.193	.847
Weight (kg)	68.4 ± 11.2	69.1 ± 10.7	*t* = 0.309	.758
Systolic blood pressure (mm Hg)	128.6 ± 11.4	130.1 ± 12.2	*t* = 0.680	.498
Diastolic blood pressure (mm Hg)	78.3 ± 8.7	79.6 ± 9.1	*t* = 0.723	.471
Heart rate (beats/min)	76.2 ± 6.9	75.8 ± 7.4	*t* = 0.285	.776
Smoking history (%)	23.1% (12/52)	26.0% (13/50)	χ^2^ = 0.105	.746
Hypertension (%)	36.5% (19/52)	38.0% (19/50)	χ^2^ = 0.020	.888
Diabetes mellitus (%)	9.6% (5/52)	12.0% (6/50)	χ^2^ = 0.133	.716
Hyperlipidemia (%)	28.8% (15/52)	32.0% (16/50)	χ^2^ = 0.134	.714
Disease duration (mo)	8.2 ± 3.1	8.5 ± 2.8	*t* = 0.534	.594
24-h proteinuria (g)	6.8 ± 1.9	7.1 ± 2.1	*t* = 0.780	.438
Serum albumin (g/L)	25.7 ± 4.3	24.9 ± 4.6	*t* = 0.942	.348
eGFR (mL/min/1.73 m^2^)	89.2 ± 14.7	87.5 ± 13.9	*t* = 0.642	.522
Positive anti-PLA_2_R antibody (%)	86.5% (45/52)	84.0% (42/50)	χ^2^ = 0.129	.719
Anti-PLA_2_R antibody titer (RU/mL)	112.5 ± 38.9	117.8 ± 35.4	*t* = 0.739	.462

CTX = cyclophosphamide, eGFR = estimated glomerular filtration rate, PLA_2_R = M-type phospholipase A_2_ receptor, RTX = rituximab.

### 3.2. Comparison of clinical remission rates

At 6 months after treatment initiation, the overall remission rate (CR + PR) in the RTX group was 73.1% (38/52), including 28.8% (15/52) achieving CR and 44.2% (23/52) achieving PR. In contrast, the CTX group showed an overall remission rate of 54.0% (27/50), with 20.0% (10/50) in CR and 34.0% (17/50) in PR. The difference in total remission rate between the 2 groups was statistically significant (χ^2^ = 4.14, *P* = .042).

At 12 months, the overall remission rate in the RTX group further increased to 84.6% (44/52), with 42.3% (22/52) achieving CR and 42.3% (22/52) achieving PR. In comparison, the CTX group achieved a total remission rate of 66.0% (33/50), comprising 28.0% (14/50) with CR and 38.0% (19/50) with PR. The intergroup difference remained statistically significant (χ^2^ = 4.84, *P* = .028).

**Figure 1.**
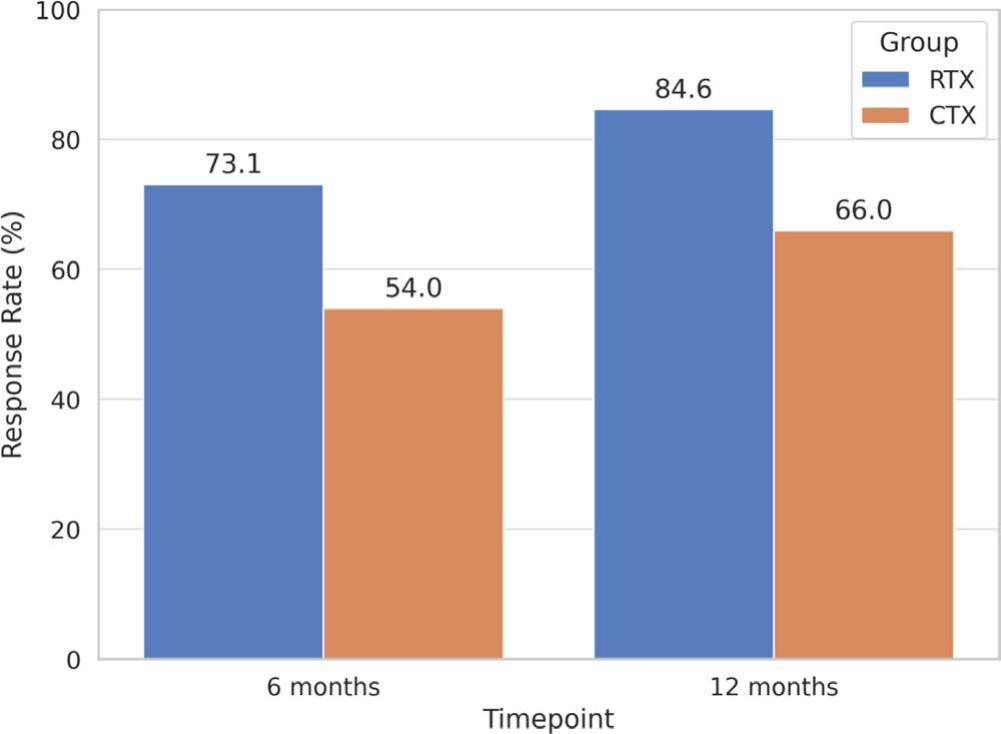
Total remission rates of RTX and CTX groups at 6 and 12 mo. CTX = cyclophosphamide, RTX = rituximab.

**Figure 2.**
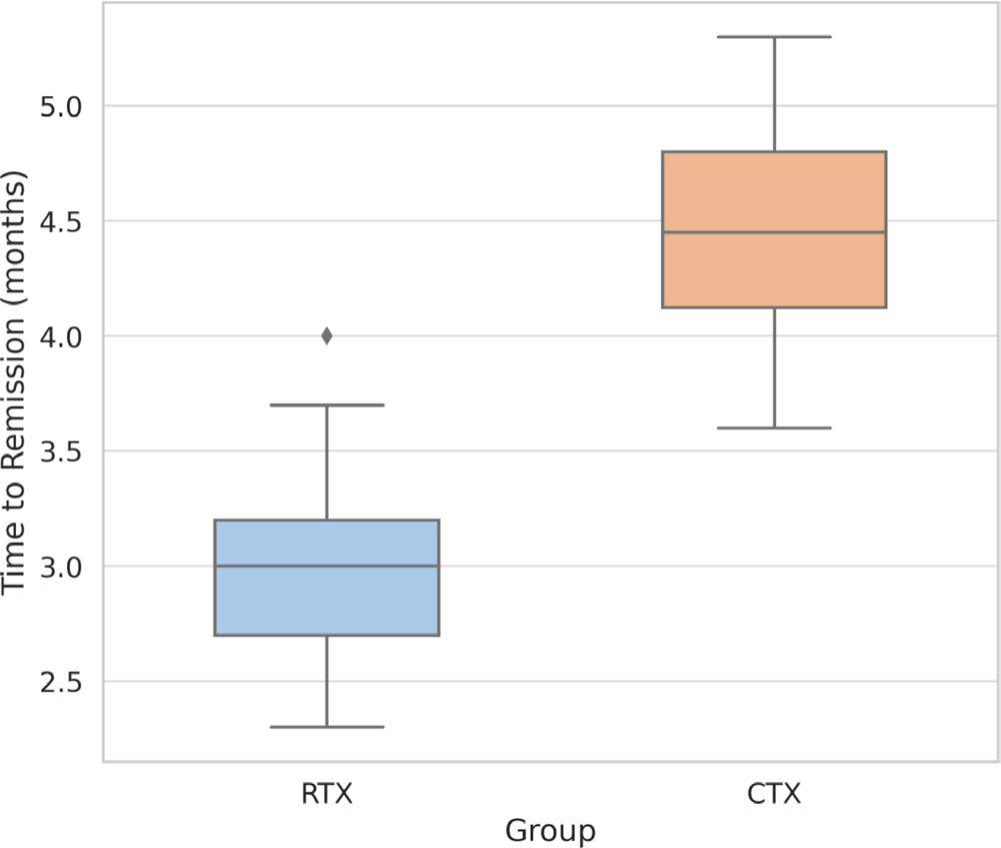
Time to clinical remission in RTX and CTX groups. CTX = cyclophosphamide, RTX = rituximab.

Furthermore, the median time to remission in the RTX group was 3.1 months (IQR: 2.3–4.0), which was significantly shorter than that in the CTX group (4.5 months, IQR: 3.4–6.1; *Z* = –2.54, *P* = .011), suggesting that RTX-based therapy led to an earlier clinical response. Detailed results are presented in Table 2 and Figures 1 and 2.

**Table 2:** Comparison of clinical remission rates at 6 and 12 mo.

Timepoint	Type of remission	RTX group (n = 52)	CTX group (n = 50)	Test statistic (χ^2^/*Z*)	*P*-value
6 mo	Total remission	73.1% (38/52)	54.0% (27/50)	χ^2^ = 4.14	.042
	Complete remission	28.8% (15/52)	20.0% (10/50)	χ^2^ = 1.10	.294
	Partial remission	44.2% (23/52)	34.0% (17/50)	χ^2^ = 1.07	.301
12 mo	Total remission	84.6% (44/52)	66.0% (33/50)	χ^2^ = 4.84	.028
	Complete remission	42.3% (22/52)	28.0% (14/50)	χ^2^ = 2.49	.114
	Partial remission	42.3% (22/52)	38.0% (19/50)	χ^2^ = 0.22	.636
Time to remission (median, months)	–	3.1 (IQR: 2.3–4.0)	4.5 (IQR: 3.4–6.1)	*Z* = −2.54	.011

CTX = cyclophosphamide, RTX = rituximab.

### 3.3. Relapse rate and sustained remission analysis

Among patients who achieved remission, 4 cases (9.1%, 4/44) in the RTX group experienced relapse during the 12-month follow-up period, all of which occurred in patients with PR. The relapses were mainly observed between 6 and 10 months after treatment. In comparison, 7 cases (21.2%, 7/33) relapsed in the CTX group, including 3 after CR and 4 after PR. Although the difference in relapse rates between the 2 groups did not reach statistical significance (χ^2^ = 3.00, *P* = .084), the RTX group exhibited a clear trend toward a lower relapse incidence.

**Figure 3.**
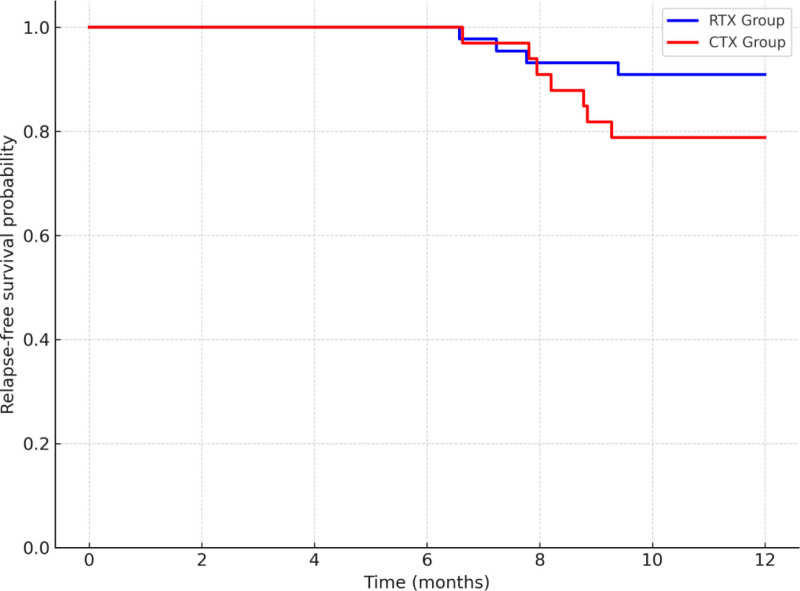
Kaplan–Meier analysis of relapse-free survival in RTX and CTX groups. CTX = cyclophosphamide, RTX = rituximab.

Regarding sustained remission, the RTX group demonstrated a 12-month sustained remission rate of 90.9% (40/44), which was higher than that of the CTX group (78.8%, 26/33). Kaplan–Meier survival analysis further revealed that the relapse-free survival rate was significantly higher in the RTX group than in the CTX group (Log-rank test: χ^2^ = 4.01, *P* = .045), indicating that RTX-based therapy may offer superior durability of remission compared with CTX. Detailed results are presented in Table 3 and Figure 3.

**Table 3:** Relapse rate and sustained remission in 2 groups during 12-mo follow-up.

Variable	RTX group (n = 44, responders)	CTX group (n = 33, responders)	Test statistic (χ^2^)	*P*-value
Relapse rate (%)	9.1% (4/44)	21.2% (7/33)	χ^2^ = 3.00	.084
Relapse after CR (n)	0	3	–	–
Relapse after PR (n)	4	4	–	–
Sustained remission (%)	90.9% (40/44)	78.8% (26/33)	χ^2^ = 2.99	.084

CR = complete remission, PR = partial remission, RTX = rituximab.

### 3.4. Anti-PLA_2_R antibody clearance and its correlation with clinical remission

Among patients who tested positive for anti-PLA_2_R antibodies at baseline, the antibody clearance rate in the RTX group reached 59.5% (26/43) at 6 months and further increased to 78.3% (34/43) at 12 months. In contrast, the CTX group showed clearance rates of 38.1% (16/42) and 56.0% (23/41) at the corresponding time points. The intergroup difference at 12 months was statistically significant (χ^2^ = 4.45, *P* = .035), suggesting that RTX more effectively promotes the decline and clearance of anti-PLA_2_R antibodies than CTX.

Subgroup analysis revealed that patients who achieved antibody clearance exhibited a significantly higher clinical remission rate compared with those who did not (91.2% vs 60.5%, χ^2^ = 8.12, *P* < .01). This finding indicates a strong positive correlation between immunologic response and clinical remission, supporting the concept that immunologic remission often precedes and predicts clinical improvement in patients with PMN. The detailed comparison is presented in Table 4.

**Table 4:** Comparison of anti-PLA_2_R antibody clearance and its association with clinical remission.

Variable	RTX group (n = 43)	CTX group (n = 42)	Test statistic (χ^2^)	*P*-value
Antibody clearance at 6 mo	59.5% (26/43)	38.1% (16/42)	χ^2^ = 3.52	.061
Antibody clearance at 12 mo	78.3% (34/43)	56.0% (23/41)	χ^2^ = 4.45	.035
Clinical remission in antibody clearers	91.2% (31/34)	–	–	<.01
Clinical remission in non-clearers	60.5% (23/38)	–	–	–

CTX = cyclophosphamide, PLA_2_R = M-type phospholipase A2 receptor, RTX = rituximab.

### 3.5. Safety analysis: comparison of adverse events

During the 12-month follow-up period, the overall incidence of AEs in the RTX group was 13.5% (7/52). The most common adverse reactions included mild to moderate infusion-related reactions (n = 3), upper respiratory tract infections (n = 2), and transient mild skin rash (n = 2). All events were self-limiting or resolved after symptomatic management, without necessitating treatment discontinuation.

In contrast, the CTX group exhibited a markedly higher total incidence of AEs at 30.0% (15/50), consisting primarily of leukopenia (n = 6), gastrointestinal discomfort (n = 5), infection (n = 3), and hepatic dysfunction (n = 1). Notably, 3 cases of moderate to severe infection required temporary treatment interruption or delay, indicating a higher overall toxicity profile associated with CTX therapy.

The difference in total AE incidence between the 2 groups was statistically significant (χ^2^ = 4.17, *P* = .041), and the severity of events was greater in the CTX group. Detailed results are summarized in Table 5.

**Table 5:** Comparison of adverse events between RTX and CTX groups.

Type of adverse event	RTX group (n = 52)	CTX group (n = 50)	χ^2^ value	*P*-value
Infusion reaction	3 (5.8%)	0 (0.0%)	–	–
Upper respiratory infection	2 (3.8%)	1 (2.0%)	–	–
Rash	2 (3.8%)	0 (0.0%)	–	–
Leukopenia	0 (0.0%)	6 (12.0%)	–	–
Gastrointestinal discomfort	0 (0.0%)	5 (10.0%)	–	–
Infection (moderate/severe)	0 (0.0%)	3 (6.0%)	–	–
Liver dysfunction	0 (0.0%)	1 (2.0%)	–	–
Total adverse events	7 (13.5%)	15 (30.0%)	χ^2^ = 4.17	.041

CTX = cyclophosphamide, RTX = rituximab.

### 3.6. Renal function changes and progression assessment

Throughout the 12-month follow-up period, overall renal function remained stable in both groups. In the RTX group, the mean baseline eGFR was 89.2 ± 14.7 mL/min/1.73 m^2^, which slightly decreased to 88.6 ± 13.9 mL/min/1.73 m^2^ at the final follow-up; this change was not statistically significant (paired *t* = 0.41, *P* = .68). Similarly, in the CTX group, the baseline eGFR was 87.5 ± 13.9 mL/min/1.73 m^2^, declining marginally to 85.9 ± 14.3 mL/min/1.73 m^2^ at 12 months (*t* = 0.61, *P* = .54), showing no significant deterioration in renal function.

Notably, no patients in either group progressed to ESRD during the study period. However, one patient in the CTX group exhibited a >30% decline in eGFR, which was associated with persistent heavy proteinuria and concurrent infection. No similar cases were observed in the RTX group. Overall, patients in the RTX group demonstrated a stable renal function trajectory during the 12-month observation period; however, the current follow-up duration is insufficient to determine long-term renal survival or sustained renal protection. The comparative results are summarized in Table 6.

**Table 6:** Comparison of eGFR changes before and after treatment in the 2 groups.

Group	Baseline eGFR (mL/min/1.73 m^2^)	12-mo eGFR (mL/min/1.73 m^2^)	*t* value	*P*-value
RTX (n = 52)	89.2 ± 14.7	88.6 ± 13.9	0.41	.68
CTX (n = 50)	87.5 ± 13.9	85.9 ± 14.3	0.61	.54

CTX = cyclophosphamide, eGFR = estimated glomerular filtration rate, RTX = rituximab.

## 4. Discussion

PMN is one of the most common pathological types of adult nephrotic syndrome, and its pathogenesis is closely associated with glomerular injury mediated by immune complex deposition. The discovery of the phospholipase A_2_ receptor (PLA_2_R) antibody has redefined PMN as a glomerular disease driven by autoimmune mechanisms.^[23,24]^ Traditionally, glucocorticoids combined with CTX have been widely used and shown to induce remission in a subset of patients. However, this regimen is often limited by its slow onset, high relapse rate, and significant adverse effects, making it less suitable for long-term disease management. Rituximab, a monoclonal antibody targeting cluster of differentiation 20, depletes B cells to inhibit the production of anti-PLA_2_R antibodies, thereby suppressing immune complex formation at its source. This represents a paradigm shift in the treatment of MN. In recent years, multiple studies have demonstrated that RTX has potential advantages in reducing proteinuria, promoting remission, and improving prognosis, although its efficacy, antibody clearance kinetics, and long-term safety vary across different populations.^[25,26]^ Based on real-world clinical data, this study systematically compared the efficacy, safety, and immunologic effects of RTX combined with glucocorticoids versus CTX monotherapy in patients with PMN, providing new evidence for optimizing therapeutic strategies.

A total of 102 biopsy-proven PMN patients with complete follow-up data were included in this study. Baseline characteristics were well matched between groups, with no significant differences in demographics, renal function, immune profiles, or comorbidities, ensuring the reliability of comparisons. Compared with previous retrospective studies, this cohort had a relatively larger sample size and excluded patients previously treated with immunosuppressants, thereby minimizing confounding from prior therapies. This design allowed for a more objective evaluation of the real-world efficacy of different initial treatment strategies in treatment-naïve PMN patients.

The results demonstrated that RTX combined with glucocorticoids achieved higher remission rates in a shorter time frame. At 6 months, the total remission rate in the RTX group was significantly higher than that in the CTX group, and the difference widened further at 12 months. This trend is consistent with findings from recent international clinical trials, indicating that RTX confers a significant advantage in achieving early clinical remission.^[27,28]^ Mechanistically, RTX depletes antibody-producing B cells, rapidly reducing circulating immune complexes, while the concurrent use of glucocorticoids accelerates the resolution of inflammatory activity. In contrast, CTX exerts its immunosuppressive effect through cytotoxic inhibition of proliferating cells, resulting in a slower impact on antibody levels and delayed overall response.

Regarding remission maintenance, this study observed a lower relapse rate in the RTX group compared with the CTX group. Although the difference did not reach statistical significance, Kaplan–Meier survival analysis revealed a significantly higher relapse-free survival rate in the RTX group, suggesting a greater potential for long-term disease stability. Previous studies have proposed that RTX maintains remission by persistently suppressing memory B cells and short-lived plasma cells, thereby delaying immune system reconstitution and preventing recurrent autoantibody production. The present findings align with this mechanism, implying that RTX may reduce disease fluctuation and enhance remission durability in long-term management. Clinically, this translates to fewer treatment adjustments, improved adherence, and enhanced quality of life for patients receiving RTX-based therapy.

The immunologic analysis further elucidated the underlying mechanism of the observed therapeutic differences. Rituximab achieved higher anti-PLA_2_R antibody clearance rates at both 6 and 12 months compared with CTX, and patients who achieved antibody clearance had significantly higher clinical remission rates than non-clearers. This finding confirms the close association between antibody reduction and clinical remission and supports the concept that immunologic remission precedes clinical recovery. Unlike many prior studies focusing solely on clinical outcomes, this study concurrently monitored immunologic and clinical responses, establishing anti-PLA_2_R antibody kinetics as an important biomarker for treatment evaluation and prognosis. The dual-dimensional analysis of immune and clinical responses represents a key innovation, providing a basis for individualized monitoring and therapeutic adjustment in PMN management.

In terms of safety, the RTX-based regimen demonstrated superior tolerability. The RTX group primarily experienced mild infusion reactions and transient rashes, which resolved spontaneously or with minimal symptomatic treatment, whereas the CTX group exhibited a higher incidence and greater severity of AEs, including leukopenia, infection, and gastrointestinal disturbances, with some cases requiring treatment interruption. These findings reinforce the favorable safety profile of RTX. Given that PMN often necessitates prolonged immunosuppression, drug tolerability directly impacts patient adherence and long-term outcomes. Reducing treatment-related toxicity not only minimizes complications but also provides a safer foundation for chronic disease management.

Regarding renal outcomes, both groups maintained stable eGFR levels during the 12-month follow-up, although the decline in the RTX group was slightly smaller, and no patient exhibited significant renal deterioration. This may be attributed to RTX’s more effective control of proteinuria and immunologic inflammation, thereby reducing glomerular hyperfiltration injury. Although the current follow-up period was insufficient to assess long-term nephroprotection fully, these short-term findings suggest a potential advantage of RTX in preserving renal function, warranting further validation in longer-term studies.

Overall, this study indicates that RTX combined with glucocorticoids not only achieves higher and faster remission but also reduces relapse rates and improves safety compared with traditional CTX therapy in PMN. The novelty of this study lies in 3 aspects: it is based on real-world data and demonstrates the efficacy and feasibility of RTX in a Chinese population; it incorporates dynamic monitoring of anti-PLA_2_R antibodies, revealing a direct correlation between immunologic and clinical remission; and it comprehensively evaluates efficacy, safety, and renal function, establishing a more integrated assessment framework for therapeutic outcomes. Nonetheless, this study has limitations, including its retrospective design, relatively short follow-up, and limited sample size. Therefore, multicenter, prospective randomized controlled trials are warranted to further substantiate these findings.

Despite the strengths of this real-world comparative study, several limitations should be acknowledged. First, as a single-center retrospective study, the inherent selection bias and information bias cannot be completely avoided. Although baseline characteristics between groups were comparable, potential unmeasured confounders – such as subtle differences in disease severity, comorbidity burden, or physician treatment preference – may still have influenced treatment allocation and outcomes. Second, the sample size was relatively limited, especially for subgroup analyses, which may have reduced the statistical power to detect small but clinically meaningful differences. Third, although anti-PLA_2_R antibody status was included as an immunological indicator, the present study did not perform a stratified analysis comparing treatment responses between PLA_2_R-positive and PLA_2_R-negative subgroups. Given that seronegativity may represent heterogeneous immunopathological mechanisms, future studies with larger sample sizes should further explore this aspect of disease heterogeneity. Fourth, the follow-up duration was only 12 months. This relatively short observation period limits the ability to assess long-term outcomes, including renal survival, relapse patterns beyond the first year, and late-onset AEs. Finally, this study did not include a pharmacoeconomic evaluation; therefore, important considerations such as the cost-effectiveness of RTX versus CTX could not be determined. These limitations highlight the need for future multicenter, prospective studies with longer follow-up and comprehensive economic assessments to validate and extend the present findings.

While RTX demonstrated favorable remission outcomes and stable renal function within 12 months, the follow-up duration of this study is insufficient to determine long-term renal survival, relapse durability, or sustained renal protection. Therefore, the observed renal function stability should be interpreted as a short-term finding rather than evidence of extended renal protection. Likewise, although RTX appears to be a promising therapeutic option based on real-world short-term data, its long-term position in treatment strategies requires further confirmation through prospective multicenter studies with extended follow-up.

## 5. Conclusion

This real-world retrospective study systematically evaluated the efficacy, safety, and immunological responses of RTX combined with glucocorticoids versus CTX in the treatment of PMN. The results demonstrated that RTX significantly improved the clinical remission rate and shortened the time to remission, indicating a distinct advantage in achieving early proteinuria control and disease stabilization. Long-term follow-up further revealed lower relapse rates and higher sustained remission rates in the RTX group, with Kaplan–Meier analysis showing superior relapse-free survival compared with the CTX group, suggesting its potential benefits in maintaining durable remission.

From an immunological perspective, RTX therapy promoted a more rapid decline and clearance of anti-PLA_2_R antibodies, and patients who achieved antibody clearance exhibited a significantly higher rate of clinical remission. This finding underscores the close association between immunologic remission and clinical improvement, highlighting the clinical value of dynamic anti-PLA_2_R antibody monitoring in guiding individualized treatment. Safety analysis confirmed that the incidence of AEs was markedly lower in the RTX group, with mostly mild and reversible reactions, demonstrating better tolerability and long-term treatment safety.

In summary, RTX combined with glucocorticoids represents a safe, effective, and mechanism-based therapeutic option for patients with PMN. By selectively depleting B cells and promoting anti-PLA_2_R antibody clearance, this regimen achieves both immunologic and clinical remission and may contribute to delaying renal function decline. To our knowledge, this is the first study to systematically validate the efficacy profile of RTX in a Chinese PMN cohort, providing important evidence for optimizing precision treatment strategies for MN. Further multicenter, long-term follow-up studies are warranted to confirm its sustained benefits and to define the optimal therapeutic regimen.

## Author contributions

**Conceptualization:** Fan Lou, Ming Yao, Hui Chen, Xueqin He.

**Data curation:** Fan Lou, Ming Yao, Hui Chen, Xueqin He.

**Formal analysis:** Fan Lou, Ming Yao, Xueqin He.

**Funding acquisition:** Fan Lou, Xueqin He.

**Investigation:** Fan Lou, Ming Yao.

**Writing – original draft:** Fan Lou, Hui Chen.

**Writing – review & editing:** Fan Lou, Hui Chen.
